# A multicentre retrospective analysis of anterior urethroplasty techniques and outcomes for urethral stricture disease secondary to transurethral resection of the prostate

**DOI:** 10.1002/bco2.70165

**Published:** 2026-05-25

**Authors:** Łukasz Białek, Mikołaj Frankiewicz, Jakub Frydrych, Marta Rydzińska, Piotr Gwara, Anna Katarzyna Czech, Piotr Chłosta, Marcin Matuszewski, Jakub Dobruch, Michał Andrzej Skrzypczyk

**Affiliations:** ^1^ Department of Urology Centre for Postgraduate Medical Education Warsaw Poland; ^2^ Department of Urology Medical University of Gdansk Gdansk Poland; ^3^ Department of Urology Jagiellonian University Medical College Krakow Poland

**Keywords:** augmentation urethroplasty, excision and primary anastomosis (EPA), iatrogenic urethral stricture, transurethral resection of the prostate (TURP), urethral stricture disease

## Abstract

**Introduction:**

Urethroplasty remains the gold standard treatment of recurrent urethral stricture disease (USD). While the literature extensively discusses urethroplasty outcomes in various contexts, there remains a need for focussed exploration into its efficacy, specifically in cases arising from transurethral resection of the prostate (TURP)‐related strictures.

**Objectives:**

To evaluate and compare different anterior urethroplasty techniques and their effectiveness in a large homogenous cohort of USD secondary to TURP.

**Materials and methods:**

A multicentre retrospective cohort study was conducted in three reconstructive urology centres in Poland, which are the referral centres for USD repair. This included patients who underwent urethral reconstruction surgery between 2015 and 2022 because of urethral stricture secondary to TURP. In all patients, the stricture was diagnosed and evaluated prior to urethroplasty by retrograde urethrography and/or voiding cystourethrography. Basic demographic and medical data including the length and localisation of the stricture, as well as details about performed urethral reconstruction and follow‐up data regarding the recurrence of the stricture and reintervention were recorded. The primary outcome was re‐intervention‐free survival after urethroplasty. Statistical analysis was performed using STATA 18 (StataCorp).

**Results:**

One hundred forty‐seven patients underwent urethroplasty because of recurrent anterior USD secondary to TURP with a mean age of 69 years. Ninety‐nine patients (67%) had a bulbar stricture, 35 (24%) had a penile urethra stricture, while 13 patients (9%) were diagnosed with penobulbar stricture. The mean length of the stricture was 24 mm, and it was significantly different among different localisations (penile – 30 mm, bulbar – 16 mm, penobulbar – 67 mm; *p* < 0.05). Half of the patients (73–50%) were treated with anastomotic urethroplasty (including transecting and non‐transecting techniques), 60 (41%) with augmentation urethroplasty, seven (5%) with substitution urethroplasty and seven (5%) underwent perineal or penile urethrostomy. Follow‐up data were available for 138 patients (94%). In the median follow‐up of 19.5 months, 19 patients (13.8%) had another procedure because of the recurrence of USD. Longer stricture length was independently associated with an increased risk of recurrence. Around 103/120 patients (85.8%) were overall satisfied with the treatment.

**Conclusions:**

USD secondary to TURP can present with various clinical manifestations, with short bulbar strictures being the most common location. The outcomes of urethroplasty are highly favourable, resulting in high levels of patient satisfaction, but do not depend on surgical technique.

## 1 INTRODUCTION

Transurethral resection of the prostate (TURP) stands as a cornerstone in the management of benign prostatic hyperplasia, providing relief to countless individuals grappling with lower urinary tract symptoms.^1^ However, this widely employed surgical intervention is not without its potential complications, and among them, urethral strictures present a challenging postoperative concern. They occur in around 3.8% of patients undergoing TURP on average, with reported incidence ranging between 2% and 10% depending on surgical technique, surgeon experience and patient‐related factors.^2^, ^3^ The onset of symptoms typically occurs within 6 to 24 months after surgery, although both earlier and delayed presentations have been documented.^4^ The aetiology of post‐TURP stricture is likely multifactorial, involving factors such as traumatic resectoscope insertion, extended resection time, ischaemia, prolonged catheterisation, infection and—increasingly recognised—intraoperative mucosal injury.^5^ Nevertheless, the primary cause of post‐TURP stricture remains unclear.^6^, ^7^ Urethral stricture disease (USD) can significantly impact the quality of life of patients and their family members and poses a challenging clinical problem because of its complex pathophysiology.^8^, ^9^

Urethroplasty remains the gold standard treatment of recurrent USD,^6^ and iatrogenic aetiology is the most common cause of USD in patients undergoing urethroplasty.^10^ While the literature extensively discusses urethroplasty outcomes in various contexts, there remains a need for focussed exploration into its efficacy specifically in cases arising from TURP‐related strictures.

This multicentre retrospective study aims to evaluate and compare different anterior urethroplasty techniques and their effectiveness in a large homogenous cohort of USD secondary to TURP and to identify variables associated with treatment failure, providing real‐world evidence to guide clinical decision‐making and improve patient outcomes.

## 2 MATERIAL AND METHODS

A multicentre retrospective cohort study was conducted in three reconstructive urology centres in Poland, which are the referral centres for USD repair. This included patients who underwent urethral reconstruction surgery between 2015 and 2022 because of urethral stricture secondary to TURP. Exclusion criteria included patients with a history of USD prior to TURP and those who had undergone previous hypospadias repair. In all patients, the stricture was diagnosed and evaluated prior to surgery by retrograde urethrography and/or voiding cystourethrography. Urethroscopy was performed in cases where diagnostic uncertainty was present. Patients who underwent DVIU or dilation only were not included in the study. The Length, Segment and Aetiology (LSE) staging system, based on stricture location and length, was applied in the analysis.^11^ We collected basic demographic and medical data, including the length and localisation of the stricture, as well as details about performed urethral reconstruction, including the type of graft used during surgery. Importantly, surgical techniques were **not standardised** across centres; instead, the choice of approach (excision and primary anastomosis [EPA]), augmentation, substitution urethroplasty or urethrostomy) was left to the discretion of the operating surgeon based on stricture characteristics, patient comorbidities and institutional preferences; however, it fulfilled the general reconstructive principles stated in the European Association of Urology guidelines on urethral strictures.^6^ All procedures were performed by four experienced reconstructive urologists who perform at least 50 urethroplasties per year. The success of the surgery was defined as improved urinary flow. In case of doubt, the evaluation with urethroscopy and/or urethrography was performed according to local protocol. We analysed follow‐up data regarding the recurrence of the stricture, defined as any need for reintervention because of USD and patients' satisfaction, collected via a structured non‐validated questionnaire.

Descriptive statistics were used to summarise demographic and clinical variables. To test the normality of variables, the Shapiro–Wilk test was used. One‐way ANOVA test was used to assess the association between the localisation and length of the stricture. Time to recurrence was analysed using Kaplan–Meier survival curves, with group differences assessed by the log‐rank test. Independent risk factors for recurrence were identified using multivariable Cox regression analysis, and the proportional hazards assumption was tested using Schoenfeld residuals. All analyses were performed with Stata 18 (StataCorp).

The study protocol was approved by the Ethical Board of Centre for Postgraduate Medical Education in Warsaw.

## 3 RESULTS

A total of 147 patients underwent urethroplasty because of recurrent anterior USD secondary to TURP in three centres between 2015 and 2022 with a mean age of 69 years. Basic patients and stricture characteristics are summarised in Table 1. Ninety‐nine patients (67%) had a bulbar stricture, 35 (24%) had a penile urethra stricture and 13 (9%) were diagnosed with penobulbar stricture. The mean length of the stricture assessed intraoperatively was 24 mm, and it was significantly different among different localisation (penile – 30 mm, bulbar – 16 mm, penobulbar – 66 mm; *p* < 0.05). Half of the patients (73; 49.7%) were treated with anastomotic urethroplasty, 60 (40.8%) with augmentation urethroplasty, seven (4.8%) with substitution urethroplasty, and seven (4.8%) underwent perineal or penile urethrostomy. Detailed information about urethroplasty techniques is presented in Table 2.

**TABLE 1:** Basic patients' and stricture's characteristics.

Patients number	147
Age (mean)	69
Diabetes	20% (29/145)
Hypertension	51% (74/145)
Suprapubic catheter	30% (44/147)
LSE stage	Available for 145
I	48% (69/145)
II	19% (28/145)
III	30% (44/145)
V	3% (4/145)

**TABLE 2:** The summary of urethroplasty techniques.

**EPA**	**73**	**49.7%**
*tEPA*	63	
*ntEPA*	10	
Augmentation	**60**	**40.8%**
*Dorsal onlay*	36	
*Ventral onlay*	17	
*Dorsal inlay*	7	
*BMG*	55	
*SlMG*	3	
*Prepuce*	3	
Substitution	**7**	**4.8%**
*Staged*	4	
*Double face*	3	
Urethrostomy	**7**	**4.8%**
*Penile*	2	
*Perineal*	5	

*Note*: The bold values are the top‐level technique categories and report the overall total *n* and percent of the full cohort treated with each category (e.g., EPA, Augmentation, Substitution, Urethrostomy). The indented non‐bold rows are subtypes that sum to the bold category total.

Abbreviations: EPA, excision and primary anastomosis; tEPA, transecting excision and primary anastomosis; ntEPA, non‐transecting excision and primary anastomosis; BMG, buccal mucosa graft; SlMG, sublingual mucosa graft.

**FIGURE 1:**
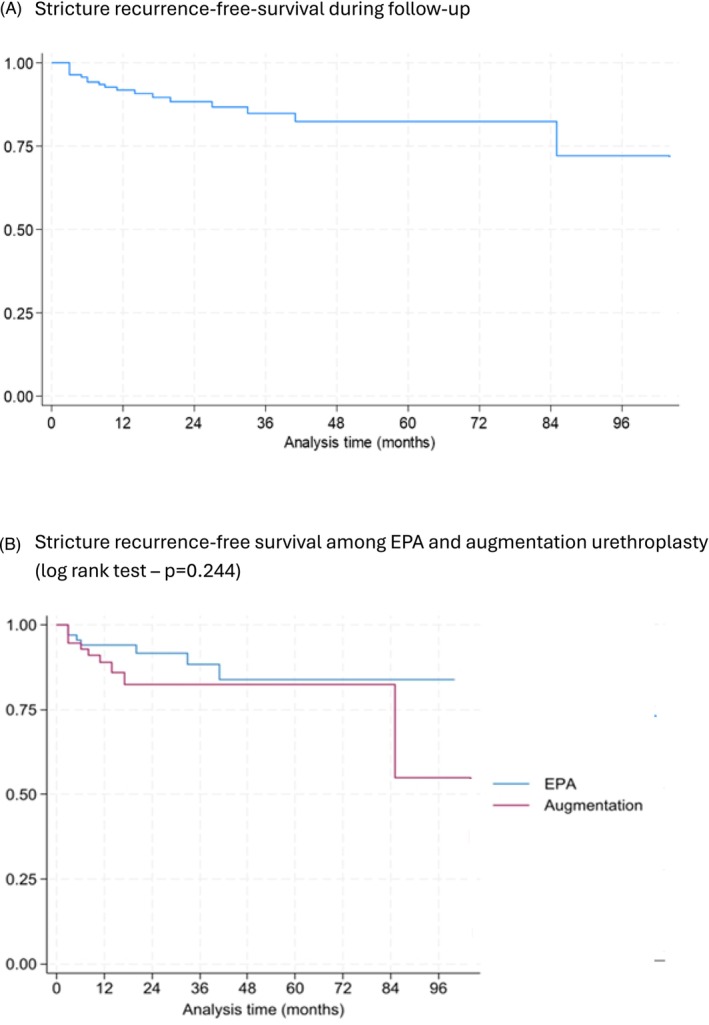
(A) Stricture recurrence‐free‐survival during follow‐up. (B) Stricture recurrence‐free survival among EPA and augmentation urethroplasty (log rank test – *p* = 0.244). EPA, excision and primary anastomosis.

Follow‐up data were available for 138 patients (94%). During a median follow‐up of 19.5 months (range 3–104 months), 19 patients (13.8%) experienced stricture recurrence (Figure 1a). The log‐rank test showed no statistically significant difference in recurrence‐free survival between EPA and augmentation urethroplasty (chi^2^ = 1.36, *p* = 0.244) (Figure 1b). Cox regression revealed that LSE stage 5 was associated with a significantly higher risk of recurrence compared to stage 1 (HR 9.89, 95% CI 2.51–38.96; *p* = 0.001). Multivariable Cox regression analysis was performed to identify independent predictors of stricture recurrence (Table 3). Longer stricture length was independently associated with an increased risk of recurrence (HR 1.03, 95% CI 1.00–1.06, *p* = 0.020). Out of 120 patients with available satisfaction data, collected via a structured non‐validated questionnaire, 103 (85.8%) reported satisfaction with their treatment outcomes.

**TABLE 3:** Multivariable cox regression analysis to identify independent predictors of stricture recurrence.

Variable	Hazard ratio	p‐value	95% CI lower	95% CI upper
Age	1.05	0.17	0.98	1.13
Diabetes	2.28	0.2	0.71	7.30
Hypertension	0.70	0.49	0.26	1.88
CAD	0.24	0.18	0.03	1.88
SPC	0.48	0.21	0.16	1.49
Localisation	1.51	0.43	0.54	4.18
Length of the stricture	1.03	0.02	1.00	1.06
Surgery type	0.64	0.35	0.26	1.61

## 4 DISCUSSION

This multicentre retrospective study evaluates outcomes of anterior urethroplasty in a large cohort of patients with USD secondary to TURP. Our findings confirm that while TURP‐related strictures predominantly affect the bulbar urethra and are generally short, they are nonetheless clinically significant and pose a substantial therapeutic challenge. Notably, we observed a 13.8% recurrence rate after urethroplasty, which aligns with reported rates in broader USD populations, suggesting that TURP‐related strictures do not confer an intrinsically worse prognosis.^11^ This recurrence rate is consistent with previously published series on post‐TURP strictures, which report rates between 10% and 20% following urethroplasty.^12^, ^13^

Importantly, we found no statistically significant difference in recurrence‐free survival EPA and augmentation urethroplasty, indicating that both approaches are viable in this subset of patients when appropriately selected.^14^ This supports a tailored surgical approach based on stricture characteristics rather than aetiology alone. However, stricture length emerged as an independent risk factor for recurrence, consistent with previous literature emphasising the prognostic importance of stricture length across aetiologies.^15^ Given the comparable recurrence‐free survival rates observed between EPA and augmentation urethroplasty in our cohort, the use of augmentation techniques may be favoured in selected cases in light of existing evidence, suggesting that non‐transecting urethroplasty with buccal mucosa grafting is associated with a lower incidence of postoperative sexual dysfunction compared to EPA.^14^

In our study, we applied the LSE system to a cohort of patients with TURP‐related urethral strictures.^16^ While it successfully identified Stage 5 as being associated with a significantly higher risk of recurrence, no significant differences were observed among Stages 1–3. This suggests that in a cohort with a homogenous iatrogenic aetiology, the discriminative performance of the LSE system may be limited, particularly in distinguishing between lower‐risk categories. However, the association between LSE Stage 5 and reintervention in this post‐TURP cohort suggests that LSE staging may be clinically actionable beyond descriptive classification. Preoperative identification of Stage 5 could improve risk counselling, inform operative planning (including anticipated reconstructive complexity) and justify closer follow‐up with earlier objective reassessment. For research, LSE Stage 5 should be prespecified for risk stratification and incorporated into multivariable prognostic models alongside patient‐ and treatment‐related factors. Prospective multicentre studies are warranted to externally validate these findings using standardised definitions (including reintervention and functional/Patient‐Reported Outcome Measure (PROM) outcomes) and to test the incremental clinical utility of LSE‐based stratification.

A substantial proportion of patients in our cohort, particularly those with shorter bulbar strictures, could be candidates for paclitaxel‐coated balloon dilation.^17^ This minimally invasive approach has shown promising results in reducing recurrence rates and improving urethral patency in selected cases of anterior urethral strictures. Incorporating paclitaxel‐coated balloon dilation into the treatment algorithm for post‐TURP strictures may offer an alternative to urethroplasty, especially for patients seeking less invasive options or those with comorbidities limiting surgical candidacy.

Patient satisfaction in our cohort was notably high, with 85.8% of patients reporting positive outcomes following urethroplasty for post‐TURP strictures. This aligns with existing literature demonstrating that successful urethral reconstruction not only improves urinary function but also enhances quality of life. Despite the challenges associated with recurrence risk, the overall satisfaction underscores the importance of incorporating patient‐centred outcomes in evaluating treatment success.^18^, ^19^

This study has several limitations. Its retrospective design may introduce selection and information biases, limiting causal inference. Although the cohort is large and homogeneous in aetiology, it is drawn from high‐volume referral centres in Poland, which may affect the generalisability to other settings or populations. The choice of the surgical technique was not predetermined but depended on the individual surgeon's preference, which may introduce a potential source of selection bias. Moreover, surgeon/centre heterogeneity was not controlled, and it may have affected the observed recurrence outcomes. Follow‐up duration, with a median of 19.5 months, might be insufficient to capture all late recurrences. The definition of success as the absence of reintervention, which—although widely used—does not fully capture the complexity of postoperative outcomes.^20^ This criterion may overlook subclinical or functional recurrences that do not prompt additional procedures. Incorporating objective functional measures and validated symptom assessments could provide a more comprehensive evaluation of treatment effectiveness. Patient satisfaction was assessed subjectively without the use of validated PROMs, which may affect the reliability of these outcomes. Finally, despite applying the validated LSE system, other potentially relevant factors influencing recurrence, such as surgical technique nuances or perioperative management, were not fully controlled.

An additional consideration is that our study includes only patients who ultimately underwent urethroplasty. In real‐world practice, initial management of post‐TURP strictures often involves endoscopic procedures (meatotomy, DVIU or dilation), and only those with recurrence or persistence are referred for reconstructive surgery. Therefore, our findings reflect outcomes in a selected population of patients requiring definitive repair rather than the entire spectrum of post‐TURP stricture disease.

In conclusion, anterior urethroplasty is an effective treatment for USD following TURP, with outcomes comparable to those for strictures of other aetiologies. The effect of the treatment is not dependent on the surgical technique. The results underscore the importance of stricture length as a determinant of prognosis and suggest that LSE Stage 5 may serve as a pragmatic preoperative marker for risk stratification, supporting individualised operative planning and follow‐up guided primarily by anatomic severity rather than aetiology. Because the study draws on a multicentre cohort limited to post‐TURP strictures only, the findings capture real‐world diversity in surgical approaches and patient care pathways, offering insight into outcomes under routine clinical conditions. Prospective studies with standardised outcome measures and longer follow‐up are warranted to validate these findings and optimise management strategies in this specific patient population.

## AUTHOR CONTRIBUTIONS

**Łukasz Białek:** Conceptualization; methodology; data curation; formal analysis; visualization; project administration; writing—original draft; writing—review and editing.**Mikołaj Frankiewicz:** Conceptualization; methodology; supervision; validation; writing—original draft; writing—review and editing. **Jakub Frydrych:** Investigation; data curation; validation; writing—review and editing. **Marta Rydzińska:** Investigation; data curation; validation; writing—review and editing. **Piotr Gwara:** Investigation; data curation; resources; writing—review and editing. **Anna Katarzyna Czech:** Investigation; data curation; validation; writing—review and editing. **Piotr Chłosta:** Supervision; resources; methodology; writing—review and editing. **Marcin Matuszewski:** Supervision; resources; methodology; writing—review and editing. **Jakub Dobruch:** Supervision; resources; methodology; writing—review and editing. **Michał Andrzej Skrzypczyk:** investigation; data curation; validation; writing—review and editing. All authors critically revised the manuscript for important intellectual content, approved the final version for submission/publication, and agree to be accountable for all aspects of the work.

## CONFLICT OF INTEREST STATEMENT

The authors declare no conflicts of interest.
